# Claims about the Misuse of Insecticide-Treated Mosquito Nets: Are These Evidence-Based?

**DOI:** 10.1371/journal.pmed.1001019

**Published:** 2011-04-12

**Authors:** Thomas P. Eisele, Julie Thwing, Joseph Keating

**Affiliations:** 1 Department of International Health and Development, Tulane University School of Public Health and Tropical Medicine, New Orleans, Louisiana, United States of America; 2 Malaria Branch, Center for Global Health, Centers for Disease Control and Prevention, Atlanta, Georgia, United States of America

## Abstract

Thomas Eisele and colleagues dispute reports in the media and elsewhere that insecticide-treated nets are not widely used, or are misused, and say that such misconceptions are not evidence-based and are damaging to malaria control efforts.

**Summary Points**

- There are a number of potentially damaging misconceptions about insecticide-treated mosquito nets (ITNs) in Africa that have been propagated in media reports, almost all of which are based on anecdotal accounts.
- While it is clear there is room for improving the level of ITN use among those who have them, and that misuse of nets occasionally occurs, we found very little evidence to support claims of widespread misuse across Africa.
- We identified only one peer-reviewed study that reported misuse of ITNs; this study was a non-probability survey of seven beaches on Lake Victoria in western Kenya, making the conclusions non-generalizable.
- Inaccurate news stories of widespread ITN misuse should be rebuked directly through the dissemination of empirical data contradicting anecdotal reports and in rebuttal editorials in newspapers and journals.

## Introduction

Nearly all experts agree that insecticide-treated mosquito nets (ITNs) are a lifesaving intervention, supported by strong evidence from carefully conducted trials that show ITNs to be efficacious at preventing all-cause child mortality and malaria morbidity in children and pregnant women [1],[2]. Under program conditions, ITNs have also been associated with significant reductions in malaria morbidity and all-cause child mortality [3]–[6].

However, there are a number of potentially damaging misconceptions about ITNs in Africa that have been propagated in media reports. One example is the recent *Los Angeles Times* article on the potential pitfalls of relying on ITNs to combat malaria across Africa: “While we see the treated nets as a lifesaving gift, they see them as a discomfort that provides only partial protection against a trivial illness. Is it any wonder that many use their nets to catch fish or as wedding veils or room dividers—all documented uses of insecticide-treated bed nets?” [7] Other examples of widespread ITN misuse have been reported by the media over the past 10 years and include claims about the use of nets as wedding veils in Uganda and Tanzania [8],[9], for fishing in Kenya and Zambia [10]–[13], as protection of plants/crops in Sierra Leone [14], as chicken coops in the Democratic Republic of Congo [15], and general misuse in Nigeria [16].

While it is clear there is room for improving the level of ITN use among those who have them [17], and that misuse of nets occasionally occurs [18],[19], we found very little evidence to support claims of widespread misuse. Unsubstantiated reports about widespread misuse of ITNs may undermine public and donor confidence in a life-saving intervention. With Global Fund replenishment pledges falling short of targets and a generally constricting donor picture, such inaccurate media articles could have the potential to do lasting damage to a global malaria control effort that is at a tipping point. While the media is often driven by negative news and controversy, when lives are at stake we should demand better.

## Claims Regarding Misuse of ITNs in Africa

The origins of these misconceptions appear to be anecdotal accounts from various sources, including community members, health workers, and individuals from non-governmental organizations and ministries of health. As can be seen in the *Los Angeles Times* article [7], one of the biggest misconceptions is that there is widespread use of nets for purposes other than protection against mosquitoes; the most commonly reported misuses appear to be fishing nets and wedding veils [7]–[16]. The other primary misconception is that individuals given ITNs refuse to use them. This would include the misconception that those who receive free ITNs frequently turn around and sell them on the open market [7],[14],[15]. Finally, there is the misconception that the ITN is a purely Western intervention that has been forced on African communities, with little regard to local norms or cultures, which has then led to their widespread rejection, misuse, or sale by recipients [7].

## The Evidence

While ITN household possession and use among children has increased dramatically since 2005 as a result of intensive investments across Africa [20], a number of cross-sectional studies prior to 2005 showed ITN use to be quite low [21]. Such studies likely contributed to the first misconception that many people given ITNs refuse to use them, especially when given out for free. However, much of the low use among children prior to 2005 was a result of households not having access to ITNs; if you do not have one, you cannot use one. A study of 15 nationally representative surveys between 2003 and 2006 showed that the biggest determinant of ITN use was intra-household access to them; the more nets a household has, the more likely a child in the house will use one [17]. This same study also showed that a third of the countries analyzed had more than 60% ITN usage among children in households possessing them; importantly, many (range 18%–70%) of the nets unused by children were being used by adults in the households. Additionally, there are cross-sectional data from Niger and Kenya that show nearly all (≥95%) ITNs received from mass free distribution are retained by the households, countering the argument that many nets distributed for free are sold or traded [22],[23]. Finally, it is important to note that in areas with high household coverage, ITNs confer protection to individuals not using them through community-level protection from reduced vector densities [24],[25].

We identified only one peer-reviewed study that reported misuse of ITNs [2]. This study was a non-probability survey of seven beaches on Lake Victoria in western Kenya, making the conclusions non-generalizable. However, data from a 2008 cross-sectional study in the Luangwa District in Zambia, a district bordered by the Luangwa and Zambezi rivers with a population heavily reliant on fishing [26], and where ITN household possession is greater than 80%, show that only 3% of households reported using their ITNs for purposes other than protection against mosquitoes (K. Macintyre, M. Littrell, J. Keating, J. Miller, T. P. Eisele, unpublished data). This is supported by findings from a qualitative study in Ethiopia that also found misuse of nets to be an uncommon problem [19].

We should remember that long-lasting insecticide-treated nets typically wear out after 2–3 years [27]. Therefore, we hypothesize that at least some of the anecdotal reports of nets being used for such things as fishing and weddings may actually be worn-out nets no longer in use for protection against mosquitoes, and thus their use for such purposes would not really constitute misuse of an effective ITN. It is critical that strategies are developed within countries to replace such worn-out nets with new, effective ITNs through keep-up campaigns [28]–[30]. It is also important to note that appropriate use of an ITN happens at night in the home and is therefore not visible in the same way that misuse of a net is for such things as fishing and weddings.

While mosquito nets impregnated with insecticides are a relatively new Western technology (circa the 1940s), many African communities have long used mosquito nets as protection against nuisance biting insects [31],[32]. However, access to mosquito nets, especially ITNs, remained appallingly low across most of Africa prior to the scale-up that began around 2005. In an attempt to rapidly achieve high coverage among vulnerable populations, the malaria control community has increasingly relied on mass distribution of free ITNs through campaigns. While it is true that mass distribution campaigns have largely been implemented in a top-down fashion, they have been shown to be more effective at rapidly achieving high and equitable coverage compared to social marketing of ITNs through the private sector [33],[34]. It is also widely recognized that social marketing of ITNs for sale at subsidized prices through the public and private sector will be needed to sustain the coverage achieved through mass free campaigns [28],[29]. As such, there has been a concerted effort by researchers to gather data on community preferences of ITN shapes, colors, and sizes to ensure they meet community preferences and maximize acceptability [19],[35],[36]. While more should be done to empower local communities to better implement malaria control interventions, the argument that ITN promotion fails to consider local cultural preferences is inaccurate.

## Next Steps

While ITN household possession and use among vulnerable populations is increasing across Africa, more should be done to continue this trend. Campaigns using community volunteers to promote use and help households hang their nets appear to be promising strategies [23],[37],[38], and should be included in national strategic plans to supplement ITN distribution campaigns. Additional research should also focus on better understanding ITN user preferences and use patterns in rural Africa, especially in areas with reported misuse of nets. This was done in the 2008 and 2010 Zambia Malaria Indicator Surveys, where questions were added to address net use patterns, preferences, and alternative uses [39],[40]. Similar questions should also be included in surveys in other countries to address this important area of research. This will then allow ITNs to be better tailored to meet consumer preferences and increase their acceptability and use.

The malaria community must also refute anecdotal rumors of widespread ITN misuse across Africa with empirical data from nationally representative household surveys that show substantial gains in ITN coverage and use. The association between reducing the malaria burden and ITN scale-up must also be highlighted to bolster existing evidence that ITNs remain a lifesaving intervention, which should remain a key tool in the arsenal against malaria in Africa.

And finally, the malaria community should demand more responsible health journalism when it comes to reporting on combating malaria in Africa. Inaccurate news stories of widespread ITN misuse should be taken on directly through rebuttal editorials in newspapers and journals, as well as through such media outlets as HealthNewsReview.org (http://healthnewsreview.org/), which aims at dispelling inaccuracies in health news reporting.

## Abbreviations

ITN: insecticide-treated mosquito net
